# Metabolomic Analysis of Biochemical Changes in the Serum and Urine of Freund’s Adjuvant-Induced Arthritis in Rats after Treatment with Silkworm Excrement

**DOI:** 10.3390/molecules23061490

**Published:** 2018-06-20

**Authors:** Tianyao Zheng, Shulan Su, Xinxin Dai, Liwen Zhang, Jin-Ao Duan, Zhen Ou-Yang

**Affiliations:** 1 Jiangsu Collaborative Innovation Center of Chinese Medicinal Resources Industrialization/National and Local Collaborative Engineering Center of Chinese Medicinal Resources Industrialization and Formulae Innovative Medicine, Key Laboratory of Chinese Medicinal Resources Recycling Utilization, State Administration of Traditional Chinese Medicine, Nanjing University of Chinese Medicine, Nanjing 210023, China; xiaogui_yaoyao@163.com (T.Z.); daixinxin1004@163.com (X.D.); 15951977078@163.com (L.Z.); 2 School of Pharmacy, Jiangsu University, Zhenjiang 212013, China; ZhenOu-yang@163.com

**Keywords:** silkworm excrement, rheumatoid arthritis, biomarker, metabolomics

## Abstract

Silkworm excrement (SE), is used as a traditional antirheumatic medicine in China. The present study was designed to investigate the therapeutic efficacy of water fraction of SE (ST) and ethanol fraction of SE (CT) at two different doses on adjuvant induced arthritis (AA) rats. Arthritis severity was evaluated by body weight, paw thickness, histological changes and index of paws oedema and spleen. Serum samples were collected for estimation of biochemical indicators and cytokines. In addition, a metabonomic method based on the ultra-performance liquid chromatography coupled with quadrupole time-of-flight mass spectrometry (UPLC-Q-TOF-MS) had been established to investigate the holistic efficacy of SE by serum and urine. Multivariate statistical approaches, such as partial least-squares discriminant analysis (PLS-DA) and orthogonal projection to latent structures squares-discriminant analysis (OPLS-DA) were built to evaluate the therapeutic effects of SE and find potential biomarkers and metabolic pathways. Administration with SE significantly ameliorated the AA severity, including body weight loss, paw swelling, histological changes and the levels of biochemical index. 33 endogenous metabolites had been identified (10 in serum and 23 in urine) in the AA rats. Urinary and serum metabolic profiling revealed that the metabolites underpin the metabolic pathway including nicotinate and nicotinamide metabolism; pentose and glucuronate interconversions; TCA cycle; beta-Alanine metabolism; purine metabolism and glycolysis or gluconeogenesis. The altered metabolites could be regulated closer to normal level after SE intervention. The results suggested SE possesses substantial anti-arthritic activity and demonstrated that metabonomics is a powerful tool to gain insight in the mechanism of SE formula in therapy.

## 1. Introduction

Rheumatoid arthritis (RA) is a chronic systemic inflammatory autoimmune disease that affects different parts of the body, inevitably causing pain, swelling and loss of function in joints [1]. RA significantly impacts quality of life, leading to severe disability in the patient. Current conventional therapies for RA patients, including disease-modifying antirheumatic drugs (DMARDs) and biologics, are not satisfactory. To the best of our knowledge, there is no unified theory about the pathogenesis of RA. Therefore, it is necessary to find an early diagnostic marker with high sensitivity and specificity.

Metabolomics is one of the newest methods focused on the association between disease and metabolic profile. Several analytical techniques have been widely used to determine the metabolites, including mass spectroscopy, ^1^H-NMR spectroscopy and liquid chromatography-mass spectroscopy [2]. Notably, emerging evidence indicates that RA susceptibility may be involved in the perturbation of metabolism [3,4]. The metabolomics approach can provide insight into the entire metabolism process and identify disparities in the metabolites and related metabolic pathways [5]. Recent evidence has also demonstrated that metabolomics approach is an effective tool in characterizing the metabolic changes of RA [6,7].

Traditional Chinese Medicines (TCMs) have been used in China for centuries and have shown efficacy in RA treatment [8]. Silkworm excrement, a classical traditional Chinese medicine, is commonly used in Chinese medicine as an anti-rheumatic drug. It was described to have the ability of “expelling wind and eliminating dampness” by Li Shizhen (during the Chinese Tang Dynasty) in his treatise “Compendium of Materia Medica” [9]. The previous study also exhibited that silkworm excrement has significant anti-inflammatory effect and analgesic effect [10]. In this study, we designed to investigate the therapeutic effect on adjuvant induced arthritis (AA) in rats and explain the metabolic mechanism of the anti-arthritic of SE by setting up an integrated platform of LC–Q-TOF-MS.

## 2. Results

### 2.1. Basic Physical Parameters Test

**Figure 1:**
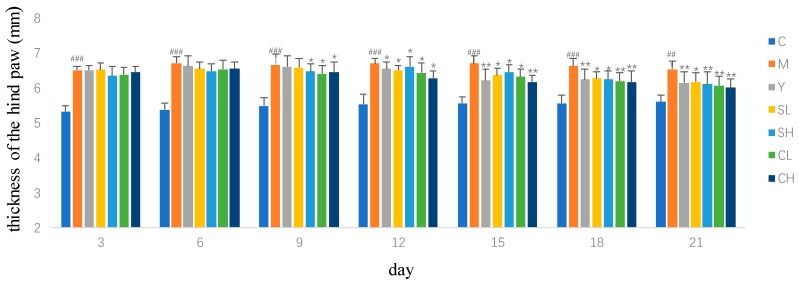
Effects of ST and CT on thickness of hind paw in AA rats. Values are presented as mean ± SD, *n* = 8. ** *p* < 0.01 compared with the AA model group; * *p* < 0.05 compared with the AA model group. ^###^ *p* < 0.001 compared with control group; ^##^ *p* < 0.01 compared with control group.

**Figure 2:**
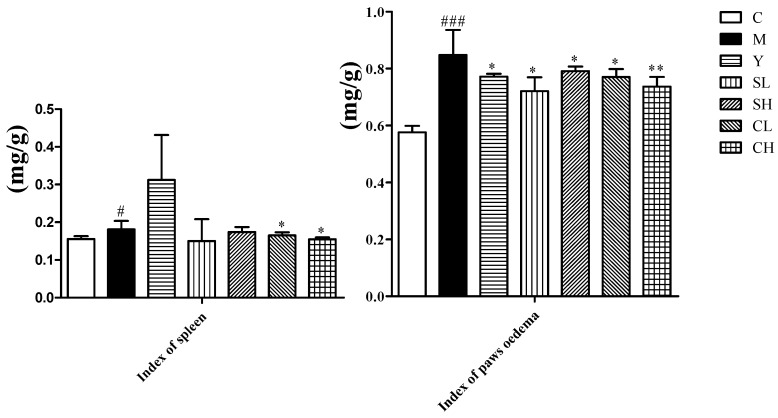
Effects of ST and CT on index of paws oedema and spleen in AA rats. Values are presented as mean ± SD, *n* = 8. ** *p* < 0.01 compared with the AA model group; * *p* < 0.05 compared with the AA model group. ^###^ *p* < 0.001 compared with the control group; ^#^ *p* < 0.05 compared with the control group.

During the experiment, body weights of control group significantly increased, while the model group increased slightly. Treatment with ST and CT could restore the body weights compared with the AA models, especially the CT groups (Table 1). In the model group, the paw swelling degrees were remarkable more serious compared with the control group. The paw swelling degrees of rats in the groups treated with ST and CT were significantly decreased compared with the AA models (Figure 1). On Day 12, the effect of CH on paw swelling degrees began to surpass the positive control group. The index of hind paw oedema and the index of spleen also exhibited an obvious recovery from model group under the treatment with CT (Figure 2). The spleen is an important immune organ. The improved effect of silkworm excrement on the spleen index indicates that it has a certain degree of immunosuppressive effect and can improve the rheumatoid arthritis symptoms by inhibiting the body’s immune response.

### 2.2. Effect of ST and CT on Histopathological Changes in Ankle Joint

**Figure 3:**
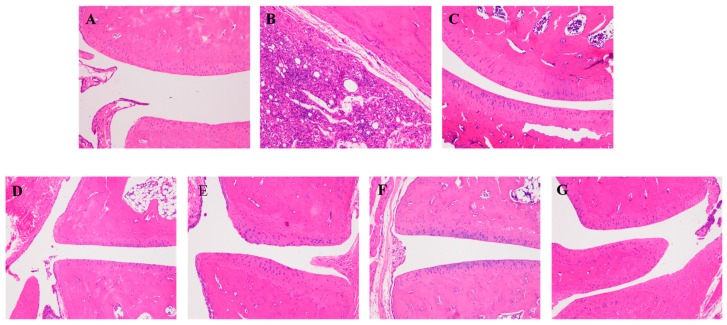
Effects of ST, CT on histopathological changes of ankle joints in AA rat (×100, HE staining). All images are from one of eight in each group. (**A**) vehicle control, (**B**) AA model, (**C**) positive control, (**D**) ST (low dose), (**E**) ST (high dose), (**F**) CT (low dose), (**G**) CT (high dose).

The ankle joints of rats in the model group exhibited synovial hyperplasia, mononuclear cell infiltration in the surrounding tissue, cartilage erosion and joint cavity narrow compared with the normal group. The AA rats received ST, CT treatment showed slightly synovial hyperplasia, prevented the infiltration of inflammatory cells and erosion of bone, and remedied joint stenosis. The rats in high dose of CT group exhibited a remarkable reduction in all pathological damages above compared with AA rats (Figure 3).

### 2.3. Effect of ST and CT on Biochemical Parameters and Cytokines of Serum in AA Rats

**Figure 4:**
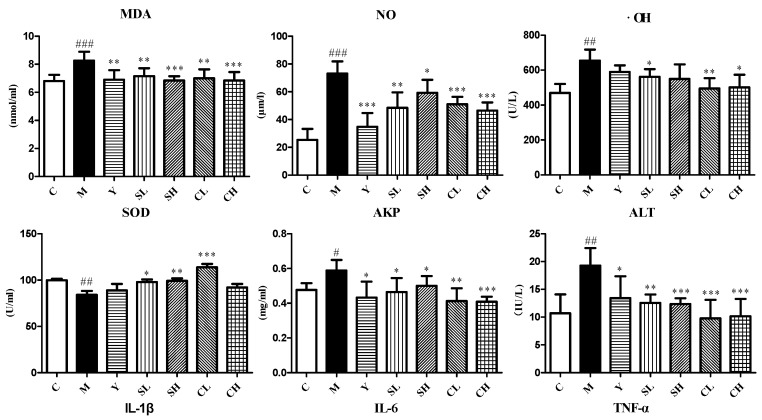

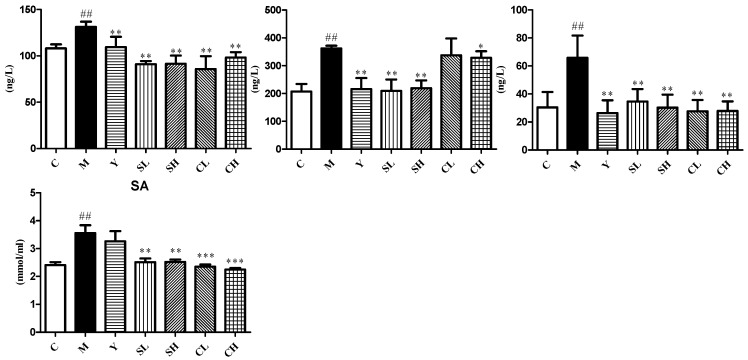
Determination of MDA, NO, ·OH, SOD, AKP, ALT, IL-1β, IL-6, TNF-α and SA in serum among all groups. Values are presented as mean ± SD, *n* = 8. *** *p* < 0.001 compared with the AA model group; ** *p* < 0.01 compared with the AA model group; * *p* < 0.05 compared with the AA model group. ^###^ *p* < 0.001 compared with the control group; ^##^ *p* < 0.01 compared with the control group; ^#^ *p* < 0.05 compared with the control group.

A significant increase in the MDA, NO and OH· levels was observed in AA rats. Meanwhile, the concentration of SOD was significantly lower in AA rats than normal animals. All these showed a higher level of oxidative stress and a lower level of antioxidant capacity. ST and CT treatments produced a significant reduction in the serum MDA, NO and OH· levels as compared to the model group, while the SOD level was also restored (Figure 4).

All the model animals showed an increase in serum AKP, ALT and SA levels. The treatments of ST, CT and indomethacin reverted all these indexes. The decreases of AKP, ALT and SA were significant compared with AA rats. The treatment of ST and CT produced a dose dependent reduction and were better than indomethacin (Figure 4).

Serum IL-6, IL-1βand TNF-α levels showed a similar effect. IL-6, IL-1βand TNF-αlevels of model group were significantly increased compared with control group. The treatment with ST, CT reverted the up-regulated levels of IL-6, IL-1βand TNF-α. While, the ST showed better effects on IL-6, IL-1βlevels (Figure 4).

### 2.4. Metabolomics Results

#### 2.4.1. QC Samples Analysis

**Figure 5:**
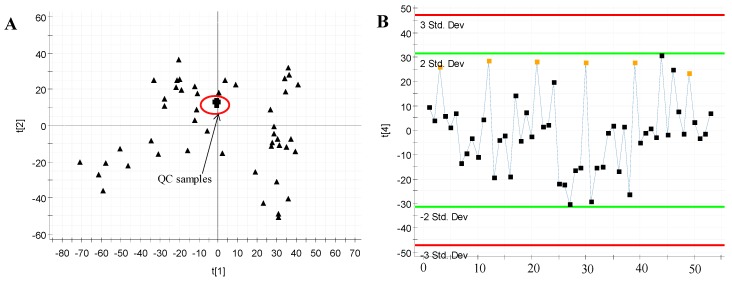
Assessment of QC samples (**A**) PCA score plot (PC1 versus PC2) of test samples and QC samples; (**B**) Trend plot showing the variation of t[1] over all observations. QC samples were colored as red boxes and test samples were colored as black triangle. *X* axis numbers represented sample number (53 injections). *Y* axis was arbitrary.

The relatively tight clustering of QC samples (Figure 5) and relative standard deviations (RSD%) of ion intensity (Table 2) demonstrated the quality of QC data. The trend plot showed the variation over all observations with respect to run order (Figure 5B). Ten ions chromatographic peaks were selected to method validation. The repeatability of method was evaluated through six replicates of QC sample. From the PCA results (Figure 5), it can be seen that the QC samples are tightly clustered together, indicating that the experimental results have little difference and the instrument stability is good. These results provided the repeatability and stability of the method were well.

#### 2.4.2. Multivariate Statistical Analysis

**Figure 6:**
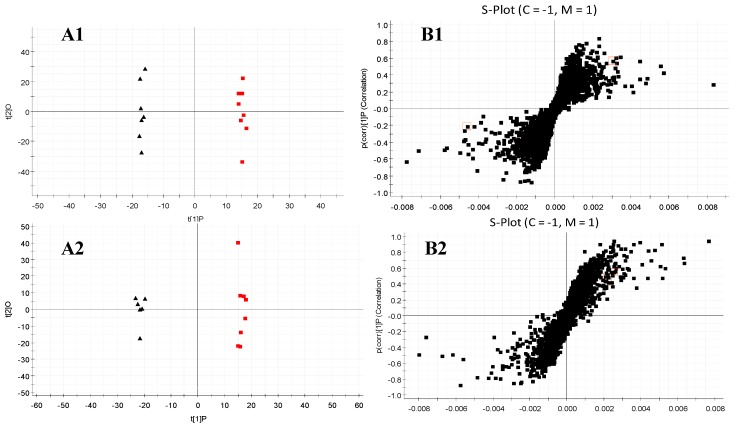

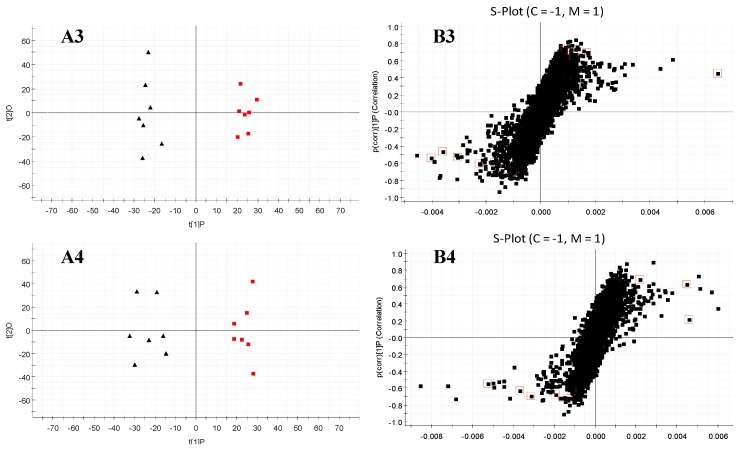
OPLS-DA scores plots (**A**) and S-plot of OPLS-DA (**B**) for serum (lable 1, 2), and urine (lable 3, 4) of AA model group (red) versus healthy controls (black) in positive and negative ion mode. The label of 1 and 3 were obtained in positive ion mode, with the label of 2 and 4 in negative ion mode. (**A1**, R^2^Y = 0.9877, Q^2^ = 0.8773; **A2**, R^2^Y = 0.9903, Q^2^ = 0.9311; **A3**, R^2^Y = 0.9835, Q^2^ = 0.8213; **A4**, R^2^Y = 0.9033, Q^2^ = 0.8433).

The data of serum and urine samples were analyzed by OPLS-DA and PLS-DA in both positive and negative modes. The score plots of OPLS-DA presented notable separation between control and model groups both in serum and urine metabolic profiles (Figure 6(A1–A4)). R2Y and Q2of the OPLS-DA model in positive and negative modes were both above 0.75; suggesting that the OPLS-DA models presented excellent classification and prediction ability. The potential metabolic markers between control and model groups could be identified from the Splot of OPLS-DA (Figure 6(B1–B4)), combining with the retention time, the standard references, precise molecular mass and MS/MS data.

**Figure 7:**
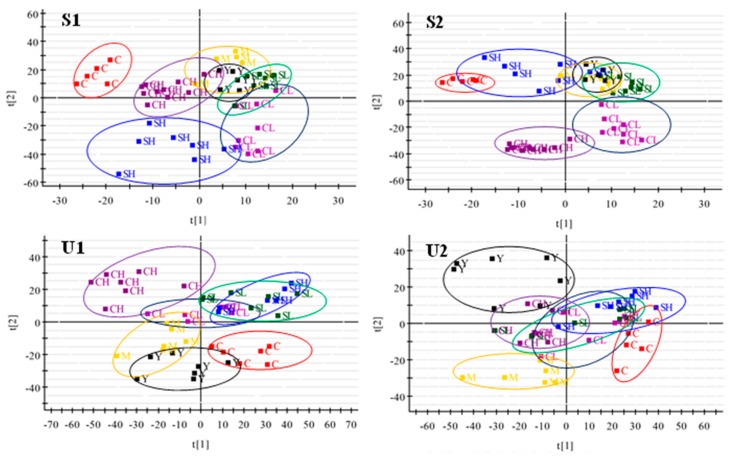
PLS-DA scores plots of serum (**S**) and urine (**U**) samples from controls, models, treatment rats in positive and negative ion mode. The label of **S1** and **U1** were obtained in positive ion mode, with the label of **S2** and **U2** in negative ion mode. (**S1**, R^2^Y = 0.9472, Q^2^ = 0.8536; **S2**, R^2^Y = 0.9773, Q^2^ = 0.9178; **U1**, R^2^Y = 0.9665, Q^2^ = 0.8923; **U2**, R^2^Y = 0.9213, Q^2^ = 0.8463).

PLS-DA model was built to exhibit the metabolic distinction among all groups (Figure 7). The R2Y and Q2 of PLS-DA model in positive and negative modes indicated that the PLS-DA model was good to fitness and prediction. The variations of metabolic profiling in serum and urine for administration group rats had the tendency to restore back to the levels of controls, especially the SH group. It was obviously found that the relative distance in positive and negative modes of SH and CH groups decreased significantly in serum compared with model group, indicating that SH and CH groups had better effects than other treated groups. While, in urine samples, SL and SH performed better.

**Figure 8:**
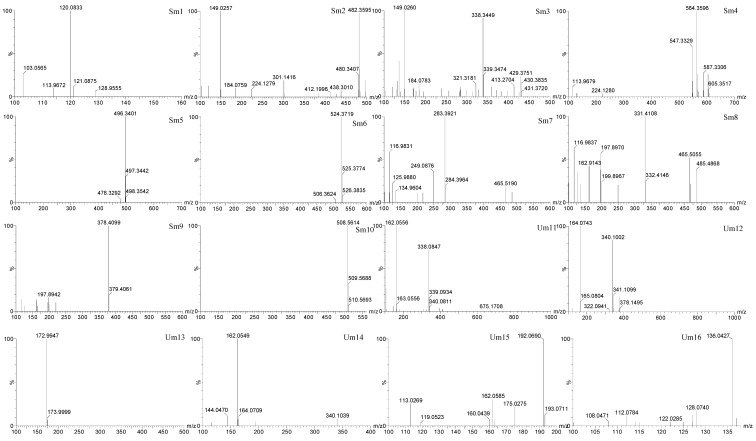

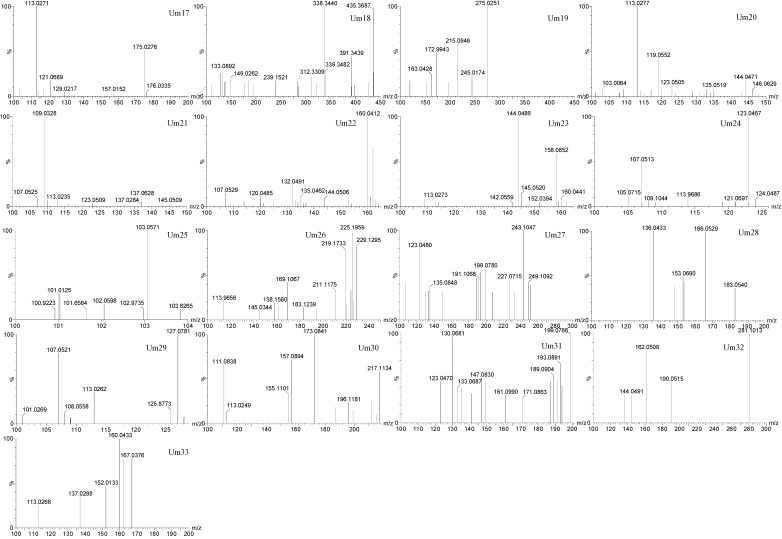
MS/MS spectra of fragment ions of metabolites.

**Figure 9:**
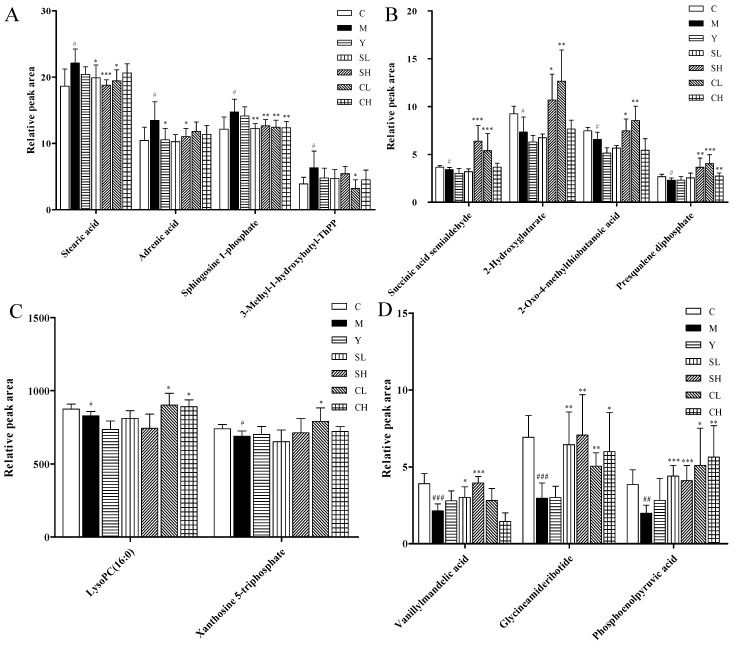

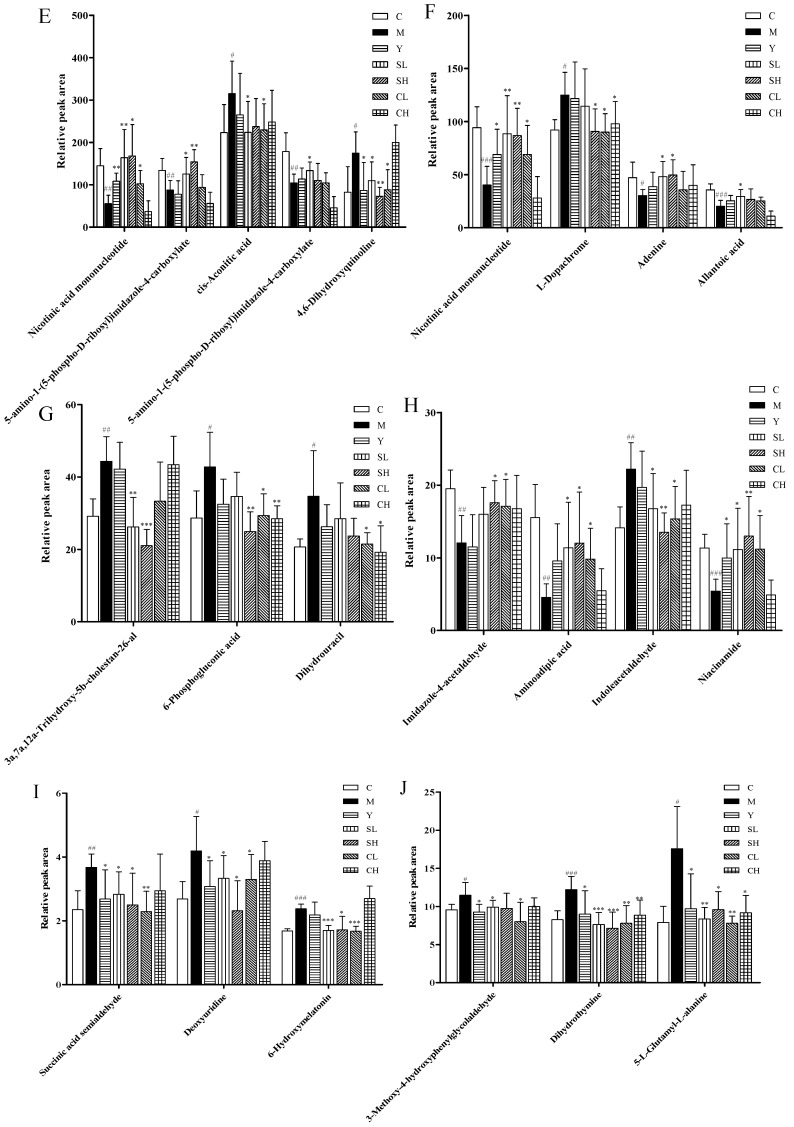
Relative peak area of potential biomarkers identified in serum (**A**–**C**) and urine (**D**–**J**) in positive and negative ion mode. Values are presented as mean ± SD, *n* = 8. *** *p* < 0.001 compared with the AA model group; ** *p* < 0.01 compared with the AA model group; * *p* < 0.05 compared with the AA model group. ^###^ *p* < 0.001 compared with the control group; ^##^ *p* < 0.01 compared with the control group; ^#^ *p* < 0.05 compared with the control group.

Identification of the endogenous biomarkers was based on retention time, mass assignment and the fragments of corresponding production for the structural identification of metabolites from the UPLC-QTOF/MS analysis platform. In this study, thirty-three endogenous metabolites (ten in serum and twenty-three in urine) were ultimately identified which showed significantly changes between controls and AA models (*p* < 0.05 or *p* < 0.01 or *p* < 0.001) (Table 3). The MS/MS spectra of fragment ions of metabolites are shown in Figure 8. After administration for three weeks, the related potential biomarkers were restored back to a control-like level. The detailed information is shown in Figure 9.

#### 2.4.3. Metabolic Pathway Analysis

**Figure 10:**
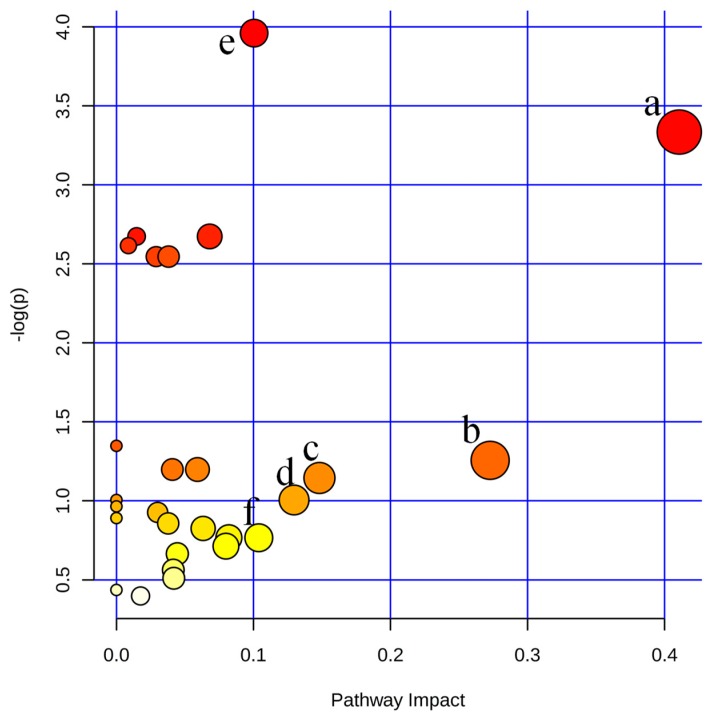
Summary of pathway analysis of serum and urine samples of rats. (**a**) Nicotinate and nicotinamide metabolism; (**b**) pentose and glucuronate interconversions; (**c**) TCA cycle; (**d**) β-alanine metabolism; (**e**) Purine metabolism; (**f**) glycolysis or gluconeogenesis.

In order to explore potential metabolic pathways affected by the treatment of ST and CT, endogenous metabolites identified above were imported into the web-based database MetPA. The pathways with an impact value above 0.10 were screened out as the potential target pathway. As shown in Figure 10, the selected metabolites are involved in nicotinate and nicotinamide metabolism, pentose and glucuronate interconversions, TCA cycle, beta-Alanine metabolism; purine metabolism and glycolysis or gluconeogenesis.

### 2.5. Pathway Enrichment Analysis

**Figure 11:**
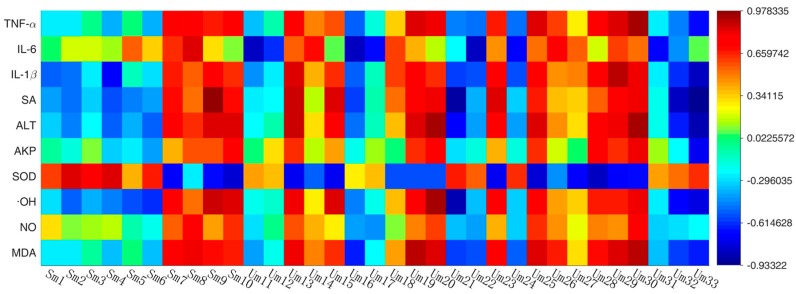
Correlation analysis between biomarkers and biochemistry indicators according to their pearson correlation coefficient. (all the labels present the same biomarkers as the Table 3).

Pearson correlation matrix analysis method was used to discover the correlations between potential biomarkers and biochemical indicators and the significance of the connection was set at the level of *p* < 0.05. The results of correlation analysis between potential biomarkers and biochemical indicators in serum and urine were presented in Figure 11.

The level of TNF-α, IL-1β, SA, ALT, AKP, ·OH, and MDA positively correlated with sm7, sm8, sm9, sm10, um13, um19, um20, um23, um29 and um30 (r > 0.6), negatively correlated with um21, um22, um32 and um33 (r < 0.6). SOD positively correlated with sm2, sm3 and sm4 (r > 0.6), negatively correlated with sm10, um13, um15, um23, um25 and um28 (r < 0.6). These correlations found in this study between biomarkers and biochemical indicators could provide reference to understand the pathological mechanisms of the RA.

## 3. Discussion

The aim of the present study was to assess the therapeutic efficacy of SE, and try to elucidate the anti-arthritic mechanism through metabolic profiling. In this study, both the ST and CT suppressed the increase of IL-1β, IL-6 and TNF-α efficiently while the ST showed better effect than CT. As previous reported, inflammation mediators such as IL-1β, IL-6 and TNF-α play an important role during the process of RA by inducing the infiltration of immune cells and stimulating the release of MMPs [11]. TNF-α is believed to be the key role in the pathogenesis of RA [12]. Many signaling pathways and pro-inflammatory cytokines relative to RA can be simulated by TNF-α [13]. IL-1 is another most important cytokine in the pathogenesis of RA, while IL-1β has more important potential to degrade cartilage and bone than TNF-α [14]. In addition, it has been demonstrated that IL-6 is likely to provide a useful adjunct to the individuals with RA who fail to respond to drugs that block TNF-α [11]. Downregulation of TNF-α, IL-1 and IL-6 simultaneously was probably impact the NF-κB signaling pathway, and relieve the symptoms of RA [15].

Meanwhile, we compared the metabolic characteristics of AA models with healthy controls by using LC-MS platforms combined with multivariate statistical analysis. To some extent, it is an effective approach to investigate how SE works. Previous metabolomics and clinical biochemistry studies have demonstrated that RA could lead to a series of complex responses from multiple metabolic pathways, such as energy metabolism, carbohydrate metabolism, lipid metabolism, amino acid metabolism and so on [16]. In this study, thirty-three metabolites were identified as potential biomarkers of AA rats, which were significantly related to six biological pathways, including nicotinate and nicotinamide metabolism, pentose and glucuronate interconversions, TCA cycle, beta-Alanine metabolism; purine metabolism and glycolysis or gluconeogenesis. The levels of adenine, aminoadipic acid, nicotinic acid mononucleotide, phosphoenolpyruvate, glycinamide, nucleotide, uric acid, Lyso-PC (16:0) were significantly decreased and the levels of succinate semialdehyde, deoxyadenosine, dihydrouracil and 6-phosphogluconate were significantly increased compared with normal controls, which were consistent with literature [17]. Lyso-PC, an important component of oxidized low-density lipoprotein (oxLDL), has been confirmed to be a chemoattractant for T lymphocytes [18]. It also induces antibody formation and macrophage stimulation. Thus the Lyso-PC could make an impact on the inflammation state of an organism. Abnormalities in these metabolites may reflect the impact of AA on phospholipid metabolic pathway. However, treatment with SE normalized these altered levels into near normal.

Notably niacin and nicotinamide metabolic were filtered out as the most important metabolic pathways in present study. Nicotinamide is an amide form of nicotinic acid which exerts its protective function against oxidative stress and inflammation by participating in the energy metabolism of cells. Furthermore, it can effectively prevent cell membranes from free radical damage and block the activation of inflammatory cells [19]. In addition, tricarboxylic acid cycle (TCA), an important energy metabolism pathway, was significantly enhanced. Abnormal excretion of TCA intermediates in AA rats may indicate mitochondrial dysfunction in RA. As cell mitochondria contains most of the tricarboxylic acid cycle enzymes, including citrate synthase (CS) [20].

Besides, glycolysis or gluconeogenesis play a key role in the upkeep of many organisms with sufficient external sugar sources. Some studies have reported that there is increased glycolytic activity in the synovial tissue of RA patients [21]. Our previous results showed that the body weight of AA models were significantly decreased compared with normal controls. By means of SE treatment, the levels of body weight could be up-regulated significantly. A previous study showed that uric acid is the metabolite of purine and pyrimidine in normal synovial tissues. It has strong anti-oxidant effect, so as to remove reactive oxygen species, directly [22]. Low concentrations of uric acid in AA models may indicate that the oxidative damage have close relationship with RA. In addition, SE exhibited significant antioxidant activities indicated by suppressive effects on MDA, ·OH and NO, up-regulation of SOD. Reactive oxygen species (ROS) are produced by normal cellular metabolism, and play an important role in the defense system against infection [23]. But in RA conditions, aggregation of activated monocyte and macrophages could result in excessive ROS production in joints and organs, and cause a direct damage to the cell membrane [24]. At the same time, the concentrations of relative enzymes such as AKP, ALT increased significantly. Administration with SE restored the metabolites and biochemistry indicators correlated with oxidative stress. The result indicated SE can protect organisms from oxidative damage.

Previous studies showed that RA can decay the uptake of amino acid. SE appeared to regulate the amino acid metabolism, mainly including tryptophan, leucine and β-alanine. Tryptophan, one of the essential amino acids in the human body, can inhibits the differentiation, proliferation and functional expression of T cells [25]. Activation of antigen-specific T cells is thought to be central to the onset and progression of RA and can accelerate joint inflammation and tissue destruction [26]. It was found that tryptophan metabolism disorders in patients with rheumatoid arthritis [27]. Leucine is known to synthesize immune-related proteins, remove aging, damaged proteins and participate in tissue repair renewal [28]. Beta-alanine, an amino acid that is not involved in protein synthesis, is widely used as a nutritional supplement to improve animal performance [29].

## 4. Materials and Methods

### 4.1. Chemicals and Instruments

Complete Freund’s adjuvant (CFA) (containing 1.0 mg of dry, heat-killed *Mycobacterium tuberculosis* (strain H37Ra) per 1.0 mL sterile, non-metabolizable oils) and the reference drug indomethacin were purchased from Sigma-Aldrich (St. Louis, MO, USA). UPLC-grade acetonitrile was purchased from Merck (Darmstadt, Germany), formic acid was purchased from Sigma-Aldrich and reference drug indomethacin was purchased from Shanghai New Yellow River Pharmaceutical Limited (Shanghai, China).

Waters Acquity TM Ultra Performance LC system (Waters, Milford, MA, USA) equipped with a Quattro Micro MS spectrometer and a Waters Xevo TM G2 QTof MS (Waters MS Technologies, Manchester, NH, USA). Deionized water was purified on a Milli-Q system (Millipore, Bedford, MA, USA). Mass Lynx v4.1 workstation was adopted to analyze the data, and Ultra-highspeed centrifuge at low temperature (Thermo Scientific, Waltham, MA, USA); DMI3000M microscope (Leica, München, Germany) were used.

### 4.2. Herbal Preparation and Extraction

The silkworm excrement (ID161104) was purchased from the Li Liang Ji Pharmaceutical Company (Suzhou, China). The silkworm excrement (5 kg) was extracted with water two times and underwent reflux extraction for 1 h each time. The two batches of filtrates were combined and concentrated through vacuum concentrating system for later research. Preparation of ethanol extract: The ethanol extract was gained through the same method. The only one thing that was different is replace water with 60% alcohol.

### 4.3. Animals

Male Sprague-Dawley rats weighing 180–220 g were purchased from the Experimental Animal Center of Zhejiang Province (Zhejiang, China; certificate no. SCXK 2014-0001). All animals were housed in cages under the controlled conditions (constant humidity: 60 ± 2%, temperature: 23 ± 2 °C, light: 2 h on/12 h off) and with standard pelleted food and water ad libitum. The animal protocol was approved by the Ethical Committee of Nanjing University of Chinese Medicine, and strictly abide by the requirement for the care and use of laboratory animals (US National Research Council, 2011).

### 4.4. Freund’s Adjuvant Induced Arthritis

All animals were allowed to acclimatize to laboratory conditions before experiment for 7 days, during which they were allowed access to food and water ad libitum. After the acclimation period, all animals were induced with 0.1 mL Freund’s complete adjuvant via intradermal immunization in rat’s right hind paw except vehicle control (with eight rats). After 7 days, all animals were induced with 0.1 mL Freund’s complete adjuvant in the base of tails to strengthen the immunity effect. Saline (0.1 mL) was injected in the right paw and tail of the vehicle control animals.

### 4.5. Administration

After the first injection, AA rats were randomly divided into six groups (M, Y, SL, SH, CL, CH) consisting of eight animals per group. According to the raw drug dose calculation of each group, the doses are as follows, SL, CL (5.4 g/kg/d), SH, CH (10.8 g/kg/d). All animals were received various doses by gastric intubation daily for 21 days. The rats of M (AA model controls) and C (vehicle controls) were given pure water instead, and the Y (positive controls) were given 3 mg/kg indomethacin at the same time.

### 4.6. Serum and Urine Sampling

After 21 days of treatment, rats were put individually in the metabolism cages for 24-h urinary collection. And the urine samples were stored at −80 °C until analysis. At day 22 (since treatment), the rats were anesthetized. The whole blood was collected through abdominal aorta, and collected into clean test-tubes with or without EDTA. Then, the blood samples without EDTA were immediately centrifuged at 3000 rpm for 10 min, and the serum samples were separated and stored at −80 °C until analysis.

### 4.7. Measurement of Paw Edema, Body Weight, Paws Oedema Index and Spleen Index

The body weight and hind paws edema were periodically measured since the second induction [30]. The intervention effect was evaluated by hind paw edema index after sacrifice. The index of hind paw edema was described as the ratio (mg/g) of hind paws weight versus body weight. After treatment was completed, the foot swelling index was used to assess the ultimate therapeutic effect of the drug on the affected foot. The index of spleen was described as the ratio (mg/g) of spleen weight versus body weight.

### 4.8. Histological Examination

After sacrificed animals, the ankle joints of the inoculated sides were harvested for analysis. The samples were stored in 10% formalin to decalcify. After two weeks, the samples were sectioned, embedded in paraffin, and sliced for hematoxylin and eosin (H&E) for general evaluation. The severity of the arthritis was evaluated based on the changes in inflammatory cells, synovial hyperplasia, pannus formation, cartilage and bone erosion [31].

### 4.9. Biochemical Analysis and Cytokines Assessment in Serum

The levels of superoxide dismutase (SOD), hydroxy radical (OH), nitric oxide (NO), malondialdehyde (MDA), alkaline phosphatase (AKP), alanine transaminase (ALT), sialic acid (SA) in serum were determined by using quantitative colorimetric assay kits according to the manufacturer instructions. The levels of interleukin-1β (IL-1β), interleukin-6 (IL-6) and tumor necrosis factor alpha (TNF-α) were determined by using rat cytokine ELISA kits according to the manufacturer instructions. All these kits were purchased from JianCheng Bioengineering Institute, (Jiangsu, China).

### 4.10. UPLC/Q-TOF/MS Analysis of Metabolic Profling

#### 4.10.1. Sample Preparation for LC−MS

The serum and urine samples were thawed at room temperature before operation. The acetonitrile (300 μL) was added into each serum sample (100 μL), then the mixture was vortexed for 60 s and centrifuged at 13,000 rpm for 10 min at 4 °C. The acetonitrile (200 μL) was added into each urine sample (200 μL), then the mixture was vortexed for 60 s and centrifuged at 13,000 rpm for 10 min at 4 °C. Prepared samples were stored at 4 °C before transferred to the autosamplers. The quality control (QC) samples were prepared by mixing 14 serum (or urine) samples randomly selected from each group (two from each group). Thus the QC samples could represent the whole sample set. The QC samples were injected every ten samples throughout the whole process to assess the method stability and repeatability.

#### 4.10.2. UPLC-Q TOF/MS Conditions

Chromatographic separation was performed on a Waters AcquityTM Ultra Performance LC system (Waters) equipped with a Waters XevoTM G2 Q/TOF-MS (Waters MS Technologies). ACQUITY UPLCTM BEH C18 column (100 mm × 2.1 mm, 1.7 m, Waters) was applied for all analyses at 35 °C. The injection volume was 2 μL and the flowrate was 0.4 mL/min. The optimal mobile phase was composed of water (A) (containing 0.1% formic acid) and acetonitrile (B). For serum analysis, the optimized UPLC elution conditions were as follows: 0~3 min, 95~55% A; 4~13 min, 55~5% A; 13~14 min, 5% A; For urine analysis, the optimized UPLC elution conditions were as follows: 0~8 min, 95~70% A; 8~11 min, 70~30% A; 11~13 min, 30~5% A; 13~14 min, 5% A.

Mass spectrometry was performed using a XevoTM G2 QTof (Waters MS Technologies), operated using an electrospray ionization (ESI) source in positive and negative mode. The ionization source conditions were as follows: capillary voltage, 3.0 kV; cone voltage 30 V; source temperature, 120 °C; desolvation temperature, 350 °C and extraction cone voltage 2 V. The cone and desolvation gas flow rates were 50 and 600 L/h, respectively. To ensure accuracy during the MS analysis via a syringe pump, Leucine-enkephalin was used as the lock mass generating an [M + H]^+^ ion (*m*/*z* 556.2771) and [M − H]^−^ ion (*m*/*z* 554.2615) in positive and negative modes, respectively. The concentration of leucine-enkephalin was 200 pg/mL and the infusion flow rate was 100 μL/min. The MS data were collected from *m*/*z* 100–1000 Da in positive and negative ion modes. The desolvation gas was set to 600 L/h at a temperature of 350 °C, the cone gas was set to 50 L/h and the source temperature was set to 120 °C. The data acquisition rate was set to 30 ms, with a 0.02 s interscan delay. The scan range was from 100 to 1000.

#### 4.10.3. Metabolomics Data Processing and Analysis

The LC–MS raw data were analyzed by Waters MassLynx v4.1 software. After data pretreatment procedures including peak finding, alignment, filtering and normalization to total area, a data matrix consisted of retention time, *m*/*z* value, and the normalized peak area was obtained [32]. The main parameters were set as follows: retention time range 1–15 min, mass range 100–1000 amu, mass tolerance 0.1 Da, and noise elimination level 5.

The multivariate data matrix was analyzed by EZinfo software 2.0 (Waters). The analysis methods containing partial least-squares discriminant analysis (PLS-DA) and orthogonal partial least-squares discriminant analysis (OPLS-DA). Prior to PLS-DA and OPLS-DA, all variables obtained from UHPLC–MS data sets were mean-centered and scaled to Pareto variance.

The quality of the model was described by the cross-validation parameter Q2 and R2Y, which represents the predictability of the model and the total explained variation for the X matrix, respectively. The variable importance in the projection (VIP) value is a weighted sum of squares of the PLS weights, reflecting the relative contribution of each X variable to the model. And the variables with VIP >1 were considered to be influential for the separation of samples in the score plots generated from PLS-DA analysis [33].

#### 4.10.4. Biomarker Identification and Metabolic Pathway Analysis

OPLS-DA was performed to discrible the metabolic difference between model group and control group. Prior to that, another OPLS-DA was performed. The normal group and the model were combined into a blank group, and all the administration combinations were the administration group. A S-Plot map between blank group and administration group was used to deleted data points that exist in administration group but do not exist in the blank group. The variables with VIP >1 in the OPLS-DA model, as well as *t*-test (*p* < 0.05) were considered as the potential biomarkers. The potential metabolites were identified according to the accurate *m*/*z*, retention time, and typical MS/MS fragment and pattern of the potential biomarkers through searching the HMDB (http://www.hmdb.ca/) databases. Pathway analysis was depended on KEGG database (http://www.genome.jp/ kegg/) and Metabo Analyst 3.0 (http://www.MetaboAnalyst.ca/), which is a web-based tool for visualization of metabolomics based on database source including the KEGG and the HMDB databases.

## 5. Conclusions

The present study validated the intervention efficacy of silkworm excrement on AA rats through many methods. Notably, we integrated information obtained from metabonomic analysis of urine and serum samples of SE-induced AA rats to characterize the systemic metabolic changes during the development of RA. Through the application of metabolomics technology, 33 endogenous metabolites (10 in serum and 23 in urine) and six significantly related metabolic pathways could be identified in the process of RA. After SE intervention, these biomarkers were restored to some extent (*p* < 0.05). Other than that, the samples were tested only at one time point, a follow-up study to verify the reproducible could be also needed in the future. Thus, the results paved the way for further elucidation of the anti-arthritis mechanism of silkworm excrement.

## Figures and Tables

**Table 1:** The results of ST and CT on body weight gain in AA rats.

Day	0	5	10	15	20
C	304.43 ± 13.83	343.33 ± 13.25	363.17 ± 15.08	378.50 ± 15.96	398.67 ± 24.14
M	290.57 ± 15.09 ^##^	320.29 ± 13.28 ^##^	341.29 ± 16.39 ^#^	353.14 ± 16.20 ^#^	368.00 ± 11.66 ^#^
Y	294.14 ± 15.26	330.14 ± 15.32	348.71 ± 15.63	364.29 ± 15.13	384.86 ± 16.60
SL	295.00 ± 18.85	332.00 ± 19.45	353.00 ± 20.22	367.11 ± 19.22	393.71 ± 20.47 *
SH	295.20 ± 13.11	328.30 ± 10.12	348.00 ± 8.46	369.00 ± 9.10	388.13 ± 13.60 *
CL	298.22 ± 15.20	334.14 ± 17.35 *	360.17 ± 13.98 *	369.67 ± 14.06 *	390.44 ± 14.99 *
CH	296.70 ± 11.75	335.00 ± 12.38 *	358.22 ± 10.28 *	370.00 ± 13.37 *	389.20 ± 12.81 **

Values are presented as mean ± SD, *n* = 8. ** *p* < 0.01 compared with the AA model group. * *p* < 0.05 compared with the AA model group. ^##^ *p* < 0.01 compared with the control group. ^#^ *p* < 0.05 compared with the control group.

**Table 2:** Coefficient of variation of ion intensity of selected ions present in the QC samples covering the range of retention times.

T_R__*m*/*z* Pairs	QC1	QC2	QC3	QC4	QC5	QC6	RSD%
1.58_218.1055	21.69666	22.13039	19.78364	19.24688	19.91056	20.79174	5.56
2.02_160.0429	21.94678	21.72565	19.82855	19.83975	19.87191	21.20539	4.84
3.01_336.0743	86.66117	88.21009	87.51105	88.2303	83.3309	86.05141	2.13
4.3_113.0266	94.79924	94.10457	94.61891	96.31335	94.83017	94.60155	0.79
5.99_417.1196	98.49249	98.61852	103.366	97.91074	104.4012	96.68017	3.17
7.48_357.1018	199.4248	198.0985	198.6274	190.8936	201.4087	194.3293	1.94
8.26_355.1405	126.9033	118.2539	121.2757	123.4014	127.2227	120.4814	2.93
9.59_291.1269	21.91479	21.79148	21.69425	22.98142	21.70019	22.18444	2.24
11.28_450.2643	11.66441	13.14806	12.26132	12.96975	13.07599	12.1941	4.79
13.24_681.2932	18.93239	20.19889	18.84712	18.31389	20.96083	18.7799	5.24

**Table 3:** Potential metabolites selected and identified between AA group and control group.

No.	T_R_/min	*m*/*z*	Formula	Identification	Vip	Trend	HMDB	Pathway
Sm1	1.23	103.0576	C_4_H_6_O_3_	Succinic acidsemialdehyde	1.25	↓	01259	Alanine, aspartate and glutamate metabolism
Sm2	8.11	149.0263	C_5_H_8_O_5_	2-Hydroxyglutarate	2.42	↓	59655	Butanoate metabolism
Sm3	13.48	149.0264	C_5_H_8_O_3_S	2-Oxo-4-methylthiobutanoic acid	1.85	↓	01553	Cysteine and methionine metabolism
Sm4	2.12	587.327	C_30_H_52_O_7_P_2_	Presqualene diphosphate	1.14	↓	01278	Steroid biosynthesis
Sm5	7.68	496.3398	C_24_H_50_NO_7_P	LysoPC(16:0)	5.44	↓	10382	Glycerophospholipid metabolism
Sm6	9.44	525.0546	C_10_H_15_N_4_O_15_P_3_	Xanthosine 5-triphosphate	3.45	↑	00293	Purine metabolism
Sm7	13.41	283.3893	C_18_H_36_O_2_	Stearic acid	2.28	↑	00827	Biosynthesis of unsaturated fatty acids
Sm8	11.94	331.4114	C_22_H_36_O_2_	Adrenic acid	1.79	↑	02226	Biosynthesis of unsaturated fatty acids
Sm9	5.97	378.408	C_18_H_38_NO_5_P	Sphingosine 1-phosphate	1.63	↑	00277	Sphingolipid metabolism
Sm10	9.44	509.753	C_17_H_29_N_4_O_8_P_2_S	3-Methyl-1-hydroxybutyl-ThPP	1.53	↑	06865	Valine, leucine and isoleucine degradation
Um11	3.02	338.086	C_11_H_16_NO_9_P	Nicotinic acid mononucleotide	11.13	↓	01132	Nicotinate and nicotinamide metabolism
Um12	3.61	340.1018	C_9_H_14_N_3_O_9_P	5-amino-1-(5-phospho-d-ribosyl)-imidazole-4-carboxylate	8.98	↓	06273	Purine metabolism
Um13	2.32	172.9933	C_6_H_6_O_6_	*cis*-Aconitic acid	4.56	↑	00072	Citrate cycle (TCA cycle)
Um14	3.7	162.0573	C_9_H_7_NO_2_	4,6-Dihydroxyquinoline	10.01	↑	04077	Tryptophan metabolism
Um15	3.67	192.0679	C_9_H_7_NO_4_	l-Dopachrome	3.59	↑	01430	Tyrosine metabolism
Um16	1.02	136.0423	C_5_H_5_N_5_	Adenine	4.89	↓	00034	Purine metabolism
Um17	6.06	175.0265	C_4_H_8_N_4_O_4_	Allantoic acid	4.86	↓	01209	Purine metabolism
Um18	13.73	435.3682	C_27_H_46_O_4_	3a,7a,12a-Trihydroxy-5b-cholestan-26-al	4.01	↑	03533	Primary bile acid biosynthesis
Um19	2.7	275.0249	C_6_H_13_O_10_P	6-Phosphogluconic acid	4.50	↑	01316	Pentose phosphate pathway
Um20	2.72	113.0266	C_4_H_6_N_2_O_2_	Dihydrouracil	1.61	↑	00076	Pantothenate and CoA biosynthesis
Um21	2.00	109.0381	C_5_H_6_N_2_O	Imidazole-4-acetaldehyde	3.25	↓	03905	Histidine metabolism
Um22	2.03	162.0579	C_6_H_11_NO_4_	Aminoadipic acid	3.98	↓	00510	Lysine degradation
Um23	2.4	158.0841	C_10_H_9_NO	Indoleacetaldehyde	3.45	↑	01190	Tryptophan metabolism
Um24	5.99	123.0475	C_6_H_6_N_2_O	Niacinamide	3.89	↓	01406	Nicotinate and nicotinamide metabolism
Um25	5.65	103.0575	C_4_H_6_O_3_	Succinic acid semialdehyde	1.01	↑	01259	Butanoate metabolism
Um26	10.05	229.1286	C_9_H_12_N_2_O_5_	Deoxyuridine	1.79	↑	00012	Pyrimidine metabolism
Um27	6.05	249.1148	C_13_H_16_N_2_O_3_	6-Hydroxymelatonin	1.24	↑	04081	Tryptophan metabolism
Um28	1.04	183.054	C_9_H_10_O_4_	3-Methoxy-4-hydroxyphenylg lycolaldehyde	2.37	↑	04061	Tyrosine metabolism
Um29	4.04	127.0788	C_5_H_8_N_2_O_2_	Dihydrothymine	1.79	↑	00079	Pyrimidine metabolism
Um30	4.52	217.1098	C_8_H_14_N_2_O_5_	5-l-Glutamyl-l-alanine	1.44	↑	06248	Glutathione metabolism
Um31	6.08	199.0781	C_9_H_10_O_5_	Vanillylmandelic acid	2.10	↓	00291	Tyrosine metabolism
Um32	2.53	285.0634	C_7_H_15_N_2_O_8_P	Glycineamideribotide	2.18	↓	02022	Purine metabolism
Um33	1.82	167.037	C_3_H_5_O_6_P	Phosphoenolpyruvic acid	1.23	↓	00263	Glycolysis or gluconeogenesis
